# Altered Pathway Analyzer: A gene expression dataset analysis tool for identification and prioritization of differentially regulated and network rewired pathways

**DOI:** 10.1038/srep40450

**Published:** 2017-01-13

**Authors:** Abhinav Kaushik, Shakir Ali, Dinesh Gupta

**Affiliations:** 1 Translational Bioinformatics Group, International Centre for Genetic Engineering and Biotechnology, New Delhi 110067, India; 2 Department of Biochemistry, Jamia Hamdard, Deemed University, New Delhi 110062, India

## Abstract

Gene connection rewiring is an essential feature of gene network dynamics. Apart from its normal functional role, it may also lead to dysregulated functional states by disturbing pathway homeostasis. Very few computational tools measure rewiring within gene co-expression and its corresponding regulatory networks in order to identify and prioritize altered pathways which may or may not be differentially regulated. We have developed Altered Pathway Analyzer (APA), a microarray dataset analysis tool for identification and prioritization of altered pathways, including those which are differentially regulated by TFs, by quantifying rewired sub-network topology. Moreover, APA also helps in re-prioritization of APA shortlisted altered pathways enriched with context-specific genes. We performed APA analysis of simulated datasets and p53 status NCI-60 cell line microarray data to demonstrate potential of APA for identification of several case-specific altered pathways. APA analysis reveals several altered pathways not detected by other tools evaluated by us. APA analysis of unrelated prostate cancer datasets identifies sample-specific as well as conserved altered biological processes, mainly associated with lipid metabolism, cellular differentiation and proliferation. APA is designed as a cross platform tool which may be transparently customized to perform pathway analysis in different gene expression datasets. APA is freely available at http://bioinfo.icgeb.res.in/APA.

Identification and characterization of biologically active or perturbed pathways is important to understand unique and significant changes in different cellular states. Recently, such pathway-centric approaches are found to be more reliable for development of diagnostic bio-markers as compared to gene-centric approaches^1^. Hence, a number of studies and methods have incorporated systems biology based approaches to predict functional gene sets or pathways which are true representatives of different phenotypic states. These approaches exploit differential analysis of case-control gene networks to predict active pathways in a disease or a cellular state. Currently, a variety of pathway analysis methods are available for identification of active pathways, for example ESEA or GSNCA^2^,^3^,^4^. Analyses of gene expression datasets using these methodologies strongly suggest that gene-gene interactions in pathway sub networks vary in response to different stimuli. In fact, gene networks are dynamically rewired in response to external or internal perturbations to form uniquely wired networks^5^. It has been found that differential wiring patterns in gene interaction networks allow stressed cells (e.g. cancer cells) to adapt to defined genetic or environmental perturbations^6^,^7^. For instance, yeast transcriptional regulatory network undergoes extensive rewiring in response to external environmental conditions^5^. Zhao *et al*. exploited rewiring concept to develop a disease specific gene prioritization tool^8^. Recently, we too demonstrated that gene connection rewiring is an important phenomena which drives melanoma progression from non-metastatic to metastatic stage^9^. We observed that pathway gene sets alter its connectivity profile along melanoma progression and key pathways can be predicted with intra-pathway rewiring analysis.

In general, pathway sub-network dynamics is a consequence of large-scale rewiring in transcription regulatory programs in response to a single or multiple biological perturbations such as mutations and cellular signals. Transcription Factors (TFs) act as major gene co-expression regulators, due to which any interference in TF-mediated gene regulatory mechanism may lead to disturbed and unregulated gene expressions which may cause amplified downstream consequences^10^,^11^. Hudson *et al*. have demonstrated that TFs, in response to perturbations, rewire their regulatory relation to upregulated Target Genes (TGs) while retaining their expression to baseline level^12^. These findings generate possibility that any altered pathway gene set may also have an underlying regulatory network rewiring, which may go unnoticed by conventional analysis. Hence any pathway alteration analysis must also include differentially regulated pathways in which one or more of its gene-set component is differentially regulated by its known TF under different conditions (Figure S1). The rationale behind such analyses is that TFs, despite no changes in their expression levels, are one of the major factors governing gene co-expression and simultaneously modulate expression of different TGs^13^. Hence, co-regulated gene sets, such as pathways that are differentially and extensively regulated by TFs are likely to exhibit context-dependent changes.

The currently available pathway analysis tools, such as netGSA^14^ are useful in understanding complex disease etiology on the basis of distinct gene expression profiles, however, the tools do not probe important GRN related aspects in altered pathways. Tools such as DINA^15^, GGEA^16^ and DRAGEN^17^ elucidate differentially regulated pathways, i.e. pathways in which TG(s) are found to be differentially regulated by their known/predicted TF(s). However, above-mentioned tools have limited capabilities to prioritize differentially regulated pathways. For instance, none of the tools consider analysis of regulatory and non-regulatory rewiring within a pathway sub network for prioritization of pathways and its gene components. DINA does not accept user-defined gene expression datasets and instead uses pre-defined background networks, whereas GGEA requires information about activation and inhibition mechanisms of each regulatory edge in query datasets, which unfortunately is not available for many datasets. Moreover, these tools also fail to reveal differentially regulated genes (i.e. TGs) and their regulators (i.e. TFs) in active pathway gene sets.

We also found that most of the available tools do not focus on enrichment analysis with genes of interest within predicted altered pathways. For instance, in context of disease-specific datasets, enrichment analysis of disease-specific genes can be a vital means of filtering a large number of altered pathways to eliminate the pathways with little therapeutic relevance. Methods like PIN-PageRank^18^ demonstrated significant improvement in prediction of key pathways using known disease genes. Yet none of the available tools, except PAGI^19^, exploit supervised algorithm for identification of components important for disease progression within altered pathways, though PAGI method simply labels all significant Differentially Expressed (DE) genes as disease genes. Apart from disease gene enrichment, available tools also lack features to perform pathway-specific “gene prioritization”. Such a pathway-specific prioritized list of genes is vital to suggest components of altered pathways for therapeutic targeting.

The limitations subsumed within existing approaches motivated us to develop a simple yet effective gene expression data analysis pipeline, which we have named Altered Pathway Analyzer (APA). With its gene network rewiring based pipeline, APA can identify altered pathways and predict their differential regulation using case and control gene expression datasets. APA can also enumerate causal regulatory factors (TG and TF) involved in pathway differential regulation. Moreover, APA performs gene enrichment with sample and condition-specific (for example, a disease condition) genes within altered pathways obtained from a rewired network. The tool also offers several features to perform sub-network analysis for intra-pathway specific gene prioritization using network centralities, rewiring potential and differential expression based analysis. The APA source code, example datasets and user manual are freely available at http://bioinfo.icgeb.res.in/APA.

## Results

**Figure 1:**
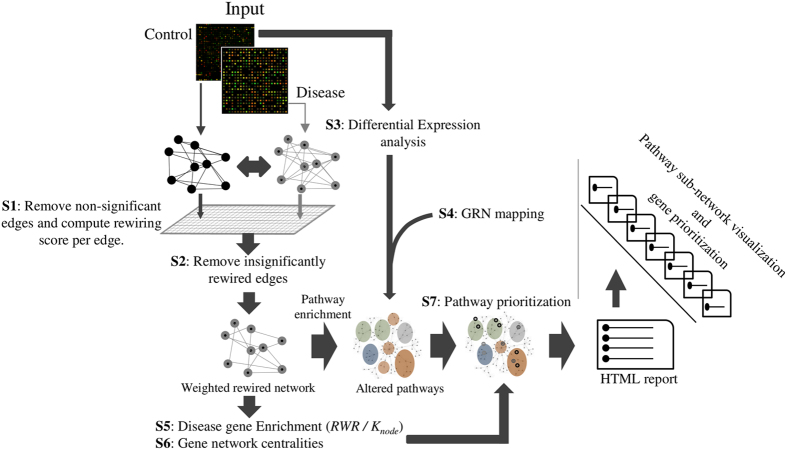
The APA workflow for elucidation of transcriptionally rewired altered pathways. The tool performs complete analysis in seven steps (S1–S7).

The APA algorithm is summarized in Fig. 1 (for details, see Materials and Methods). Initially, APA decomposes the input case and control co-expression networks to generate a single network consisting of significantly rewired edges. Next, the input pathway gene sets are mapped to the generated network structure for each pathway. The significantly enriched pathways are shortlisted for rewiring density measurement to enumerate altered pathways. Next, using the reference regulatory network, APA measures aberrant regulatory interactions between a given pathway gene and its regulator(s), i.e. known and user-defined TFs. The last step renders an enumeration of differentially regulated pathways. The tool also exploits “guilt-by-association” principle to measure disease related attributes of a pathway gene set by calculating closeness of its genes with known disease genes within a rewired network. Additionally, APA uniquely aids pathway-specific gene prioritization by measuring rewiring score and centrality of each pathway gene in a rewired network.

We validated the APA pipeline by analyzing simulated as well as real datasets which illustrates its potential for identification and prioritization of altered pathways, including differentially regulated pathways.

### Simulation study

**Figure 2:**
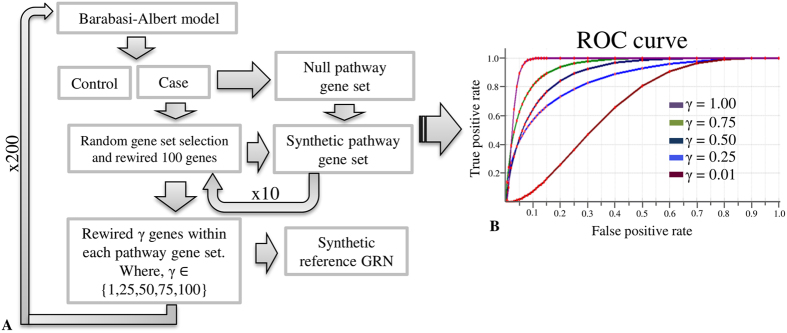
(**A**) Network simulation workflow to measure tool accuracy. (**B**) The results produced from simulated network analysis for different values of gamma. (see text).

We evaluated APA performance by identification of differentially regulated pathways under controlled simulated conditions (Fig. 2A). We simulated a network containing 2000 nodes using Barabasi-Albert model of preferential attachment^20^. A pathway set composed of 100 pathways was generated, out of which 10 pathway gene sets were labelled for differential regulation (i.e. DR pathways), while the remaining 90 gene sets were considered as null models. Each DR pathway gene set was composed of 100 genes, whereas the null model gene sets were composed of 50–100 genes. To begin the simulation, at least one connection within DR pathway members gene set was rewired. Thus, all the DR pathways were rewired, as opposed to the null model gene sets. This was achieved by creating two copies (control and case) of the simulated network and replacing strength of interaction in one copy by a random value (between 0–1). However, all intra-pathway connections in a null model gene sets were retained in case and control networks. The above-mentioned steps ensured that all differentially regulated pathways were altered too for downstream simulation.

The next step was to mimic rewired regulatory connections between the DR pathway and other network genes that were not members of the DR and null model gene sets, i.e. non-pathway genes. The edges between selected network genes and DR pathway genes were rewired by changing strength of interaction in case network. However, five different scenarios were created in which only a portion of the DR pathway genes (*γ* ∈ {0.01, 0.25, 0.50, 0.75, 1.00}) were rewired with non-pathway genes. These rewired edges were considered as regulatory rewired edges and *γ* proportion of pathway genes were considered as regulatory genes under each scenario. Finally, 10 pathway gene sets were created in which all the genes were rewired and *γ* proportions of genes were rewired with non-pathway genes across case-control networks. Two hundred replicates for each scenario were generated and APA was implemented for identification of the pathways predicted as differentially regulated (APA prediction score, *Dy* > 0). In all replicates of each scenario, 10 DR pathways were used as a true positive set and 90 null-model gene sets were used as a true negative set. The APA performance was evaluated by plotting Receiver Operating Characteristic (ROC) curves. For different *γ* values, a different area under ROC curves (auROC) was obtained that reflected the APA prediction accuracy (Fig. 2B). Clearly, as proportion of regulatory genes (*γ*) increased, APA prediction accuracy also increased. For *γ* = 0.1, we observed high false positive rate and therefore AUC was as low as 0.663; however, as the pathway differential regulation increased, prediction accuracy also increased. For *γ* ≥ 0.75, auROC exceeded 0.9, which suggests that APA was able to identify a positive test set with very high sensitivity and specificity in cases where pathway gene-set is differentially regulated. The results were as expected, as the number of rewired regulatory genes (*γ)* acting within a given pathway increased, chances of its prediction as differentially regulated also increased.

### Comparison with other tools: Pathway analysis of p53 mutated NCI-60 cell lines

In order to evaluate performance and potential of APA in identification of altered and differentially regulated pathways, we performed pathway analysis of p53 status gene expression dataset^21^. The dataset comprised of 50 NCI-60 cell line samples out of which 17 cell lines carried native p53 status and 33 cell lines carried mutated p53 status. The dataset is a popular choice for validating potential of a tool for detection of pathway level aberrations, as used in pathway analysis tools developed earlier (including the ones evaluated by us). We examined APA performance by evaluating key pathways predicted exclusively by APA, as compared to other gene set analysis tools. We compared APA with ORA^22^, GSCA^23^, GSNCA^4^, ESEA^2^, SPIA^24^, PWEA^25^ and DRAGEN^17^ for detection of altered pathways in the p53 expression dataset. The tools other than APA broadly represent diverse methodologies for the prediction of pathway perturbations- differential expression to differential co-expression; gene based to edge-based. The ease of usage, access, date of publication and citations determined our choice of the tools for comparison with APA.

**Figure 3:**
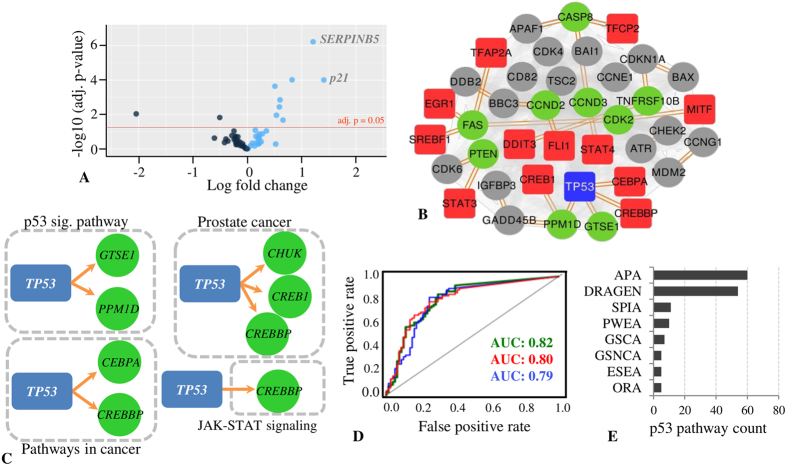
APA validation using p53 dataset. (**A**) The volcano plot to show distribution range of gene expression values vs adjusted p value in “p53 signaling pathway”. Only 10 genes which constitute ~16% of the pathway gene set were found to be DE (**B**) APA predicted p53 signaling pathway subnetwork with rewired interactions (orange color). Blue nodes present TF and red nodes represent TFs that are not an integral part of the pathway gene set. (**C**) The predicted altered interaction in top four most altered pathways predicted by APA analysis. (**D**) ROC plots representing accuracy of APA in predicting pathways with known p53 target genes. Each line represents ROC curve obtained using down-sampling the p53 mutated sample size. Green: sample size 33; red: sample size 26; blue: sample size 17. (**E**) Number of altered pathways with p53 target genes predicted by various pathway analysis tools.

In absence of any “gold-standard” outcome for pathway alteration analysis tools^3^, we first evaluated potential of different algorithms for detection of aberrations associated with KEGG “p53 signaling pathway”. The reason for choosing p53 related pathway was based on the assumption that tumor suppressor p53 mutation in the given dataset should significantly affect interaction with its direct molecular targets. The pathway was also important as p53 acts as a TF and any mutation may lead to differential regulation of its TGs. We thus expected APA to predict alteration among the intra-pathway gene set connections and differential regulation of p53 TGs in response to mutation. Although pathway genes are not over-expressed and most genes have fold change <0.50 (Fig. 3A), APA analysis suggested considerable pathway alteration associated with the p53 signaling pathway. Using the KEGG pathway database, APA predicted significant alteration (rank = 4) associated with “p53 signaling pathway” along with “pathways in cancer” (rank = 1), “JAK STAT signaling pathway” (rank = 2). However, such quite expected and APA predicted altered pathways are not detected by a majority of the other prediction tools. Except SPIA, none of the tools evaluated by us predicted the overrepresentation of p53 signaling pathway (SPIA reported p = 0.003); nevertheless SPIA failed to predict significant perturbation within pathway genes (p = 0.888; see the Supplementary File S1). Intriguingly, DRAGEN identified differential regulation associated with KEGG “p53 signaling pathway”, however, with high p-value (p = 0.943). Moreover, DRAGEN analysis did not reveal the TFs leading pathway differential regulation. The highest score from DRAGEN analysis was obtained for “pathways in cancer” (3263.700). On the contrary, APA analysis suggests significant rewiring of p53 signaling pathway sub network (Fig. 3B), wherein *CDKN1A*, an important cell cycle regulator gene, demonstrated maximum strength of local rewiring score (3.97). Another cell cycle regulator *CDK2* displayed the maximum overall centrality score (2.74) in sub-network analysis. Intriguingly, *TP53* was the only TF that belonged to the pathway gene set. In fact, most of the predicted DR pathways (~65%) in the dataset were differentially regulated with non-pathway TF(s) only. In a nutshell, the pathway was differentially regulated with 12 TFs and 9 TGs in response to the p53 mutation, which included 4 rewired connections with respect to TP53. We also measured APA robustness in prediction of expected outcome, by interchanging case and control samples. We observed that even after swapping of case-control datasets, APA could predict pathway alteration associated with p53 signaling pathway, though with a lower score (score = 0.2; rank = 8). The result signified that although gene differential expression plays a key role in prediction of pathway alteration, yet APA primarily considers other factors, such as correlational changes in order to predict pathway alterations.

It was also observed that the top four APA-predicted altered pathways includes *TP53*-mediated differential regulation of pathway genes (Fig. 3C). In “p53 signaling pathway”, “pathways in cancer” and “prostate cancer”, p53 regulator gene is an element of the pathway gene set; however, in “JAK-STAT signaling pathway”, p53 differentially regulates its TG via cross-talk. Thus, TP53 mutation has a profound effect on gene regulatory connections, which may not be taken into account and highlighted by currently available pathway analysis tools.

In search of more concrete evidences, we shortlisted KEGG pathways with at least one known TG for TP53 and considered it as a test set, while other pathways were considered as negative sets. Herein, we hypothesized that TP53 mutation may affect expressional pattern of its TGs, thus the respective pathways may be more susceptible to pathway subnetwork rewiring and differential regulation. Out of the 80 p53 target and 80 non-target pathways in test set, APA successfully predicted 60 true positives and 59 true negatives with an overall auROC of 0.82 (Fig. 3D). Moreover, down-sampling of p53 mutated samples also did not significantly alter the tool accuracy for prediction of p53 target pathways (Fig. 3D). Down-sampling was performed by randomly reducing the case-sample size by 25% till it became equal to the control-sample size, i.e. *n* = 17. We observed that even for the same number of case and control samples, APA demonstrated an auROC of 0.79. This suggests that APA prediction is robust and independent of sample size imbalance for detection of significant pathways.

Summarily, APA successfully predicted the highest number (60 out of 80) of the differentially regulated p53 target pathways (DR score > 0.05); however, the other pathway analysis tools predicted comparatively fewer altered pathways with known TP53 targets (Fig. 3E and Supplementary Figure S2). DRAGEN predicted differential regulation in only one p53 target gene set (p ≤ 0.05). However, it could identify 54 differentially regulated p53 target pathways with a score > 0, though with insignificant p value (p > 0.05). We observed that most pathway analysis methods fail to predict alteration in pathways with known p53 target genes. The results are not surprising, as all the tools compared by us, except DRAGEN, do not emphasize on regulatory edges for identification of altered pathways. Therefore, the pathway analysis tools evaluated by us are not sufficient to highlight altered pathways with TF-mediated differential regulation.

We also measured the alteration score for each APA predicted altered pathway from the 1000 shuffled case-control datasets (see Supplementary Method). The analysis revealed 10 statistically significant altered pathways (p-value ≤ 0.05; Table S1) including pathways like “Pathways in cancer” and “MAPK Signaling pathway”. The results clearly reveal the potential of APA in highlighting altered pathways using gene expression datasets.

### APA identified 366 differentially regulated pathways in pancreatic cancer

Pancreatic Ductal Carcinoma (PDC) is one of the most common pancreatic neoplasm types, with a very poor patient survival rate^26^. Numerous studies have identified a range of altered pathways involved in driving PDC progression; however, differentially regulated pathways involved in PDC are yet to be elucidated^27^,^28^,^29^. We performed APA analysis on the PDC microarray dataset (GSE28735) to determine altered and differentially regulated pathways in PDC progression. The dataset comprised of expression values corresponding to 28,869 probes in 90 samples out of which 45 samples were normal pancreatic tissue samples and the rest 45 were PDC tumor samples.

**Figure 4:**
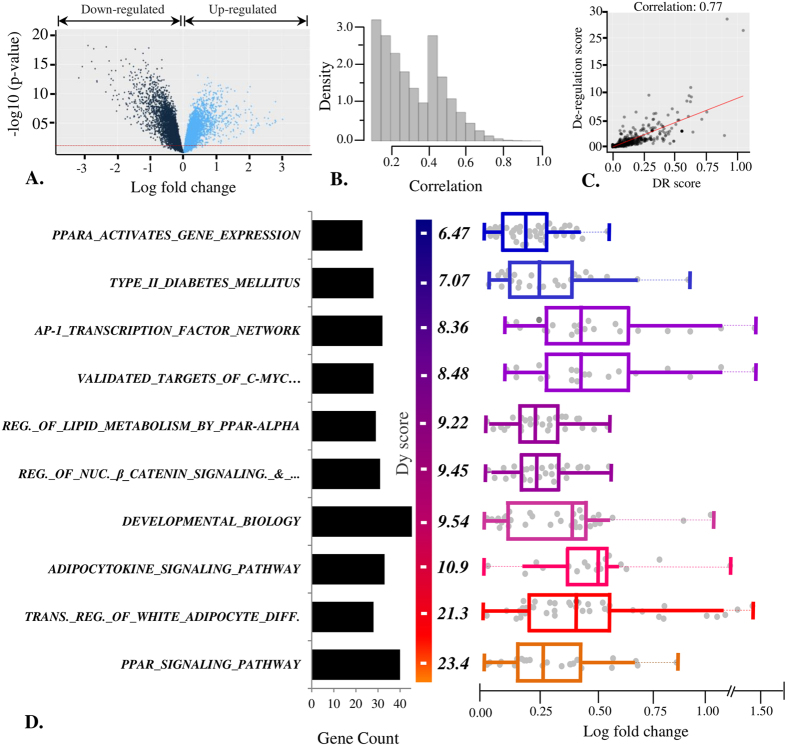
Prostate cancer dataset analysis. (**A**) Differential expression pattern of genes in PDC dataset GSE28735. (**B**) Distribution of correlation values of significantly rewired edges (p <= 0.05) in disease network. (**C**) Scatter plot representing the correlation between pathway alteration and differential regulation score (DR score) predicted in the PDC dataset. (**D**) Top 10 most altered pathways along with their gene count, alteration scores and gene differential expressions.

We began the analysis by measuring differential gene expression in the PDC dataset, which suggested a marked disruption in gene expression profile and indicates a preliminary evidence of a disrupted regulatory machinery (Fig. 4A). We observed 4205 down-regulated genes (logFC < 0 and adj. p-value ≤ 0.05) and 4179 up-regulated genes (logFC > 0 and adj. p-value ≤ 0.05). Moreover, the rewired co-expression network analysis also suggested simultaneous disruption in gene-gene interactions. The rewired network is composed of 10935 genes with 3137983 edges and correlation distribution ranging from 0.2 to 1.0 (Fig. 4B). We performed APA analysis of PDC dataset using pre-compiled pathway gene sets (see Materials and Methods). Remarkably, APA predicted 887 altered pathways out of which only ~41% (*n* = 366) are differentially regulated (with default threshold; Supplementary File S2). The complete list of altered pathways consists of several high-ranked cancer related pathways, including “prostate cancer” and “cell cycle”. We also observed strong correlation between pathway alteration and its differential regulation by variety of TFs (Fig. 4C). We analyzed top ranking altered pathways with significant intra-pathway gene set rewiring and differential regulation score. Interestingly, the top-ranked pathways are mainly composed of genes with insignificant fold changes in expression values (Fig. 4D), hence any approach based on gene differential expression may fail to highlight the important and significant features of underlying pathway alteration.

Analysis of 10 top ranking altered pathways suggest involvement of biological processes mainly linked with lipid metabolism or cellular proliferation, whereas the most differentially regulated pathways are related to regulation of adipocyte differentiation (DR score = 1.04). The results suggest significant role of lipid metabolism in prostate cancer progression. “PPAR signaling pathway” is most differentially regulated pathway gene set with a strong network rewiring. The pathway has been implicated in fatty acid oxidation and its activators have been proposed for treatment of cancer and other metabolic diseases^30^,^31^. One of the major regulators in the pathway, *PPARA* gene, is a nuclear TF superfamily protein, i.e. Peroxisome Proliferator-Activated Receptors (PPARs)^32^. A number of studies have concluded that the PPARs activation is linked with oncogenesis by induction of cell proliferation and apoptosis inhibition^31^,^33^. Sub-network analysis of the signaling pathway proves that *PPARA* gene network wiring is significantly altered in prostate cancer. This TF differentially regulates 8 of the 14 TGs, including the most rewired gene *APOC*3, an essential component of lipoprotein metabolism. Apart from *PPARA*, Retinoid X Receptor family of TFs, i.e. *RXRA, RXRB* and *RXRG*, differentially regulate a number of other TGs. Overall, the pathway is differentially regulated by 8 TFs and more than 90% of the pathway genes rewire at least one intra-pathway connection. The most central gene in the pathway sub-network is *CYP4A22*, a cytochrome P450 family gene. Products of this gene family actively participate in drug metabolism and synthesis of lipids like steroids and cholesterol^34^.

**Figure 5:**
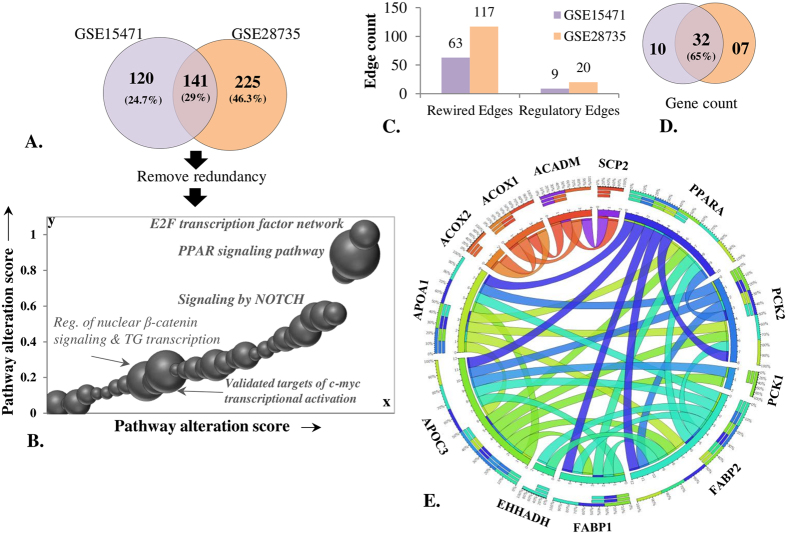
Prostate cancer dataset analysis. (**A**) Number of pathways predicted by APA analysis in two independent PDC GEO datasets. (**B**) Comparison of alteration score obtained by common pathways across different datasets (GSE28735 and GSE15471). The size of sphere corresponds to alteration score obtained in GEO dataset GSE28735. (**C**) Number of rewired and regulatory edges observed for “PPAR signaling pathway” in both datasets. (**D**) Common “PPAR signaling pathway” genes across two different sub-networks predicted from two different PDC datasets. (**E**) The sub-network representing common significantly rewired edges observed for PPAR SIGNALING PATHWAY across different dataset.

The findings motivated us to re-perform similar analysis on different PDC GEO dataset (GSE15471). APA predicted 648 altered pathways in the second PDC dataset (Supplementary File S2). As expected, the pathway list was enriched with cancer related pathways, including “E2F transcription factor network”, “signal transduction” and “cell cycle”. Intriguingly, despite differences in experimental conditions used for generating the two gene sets, 141 altered pathways were found to be common in the two (Fig. 5A). However, the list comprised of a few similar or redundant gene set terms, which were removed to retain 136 common pathways (Supplementary File S2). Surprisingly, “PPAR signaling pathway” consistently demonstrated high alteration along with several other cancer-related pathways in both the datasets (Fig. 5B). Such consistent disruption in the connectivity pattern of gene sets with similar functional role suggests a conserved mechanism that drives cancer progression despite underlying genetic and epi-genetic heterogeneity.

We also observed that though functional role of altered pathways are conserved, inherent network components and its wiring may vary across different disease samples. For example, “PPAR signaling pathway” differential regulation mediated by 6 different TFs in the first PDC dataset mediated, is not fully observed in the second dataset. We also observed approximately two-fold differences in number of rewired and regulatory edges for this signaling pathway across different datasets (Fig. 5C). Despite underlying connectivity profile differences across the two PDC datasets, altered connections of *PPARA* remain majorly unchanged, especially for targets like *APOA1, APOC3, FABP1, FABP2* and *PCK2*. Around 60% of “PPAR signaling pathway” genes remain connected even under varying experimental conditions (Fig. 5D), out of which 35 connections between 12 genes remain significantly rewired (Fig. 5E). Out of the 12 significantly rewired genes, 5 genes demonstrated a significantly higher degree of aberrations across the two datasets. The 5 genes are *FABP2, PCK2, APOA1, APOC3* and *PPARA* transcription factor. Despite limited common altered components and features, “PPAR signaling pathway” pathway is consistently predicted as significantly altered across the two conditions, with a common source of PPARA-mediated co-expressional changes. However, it is not mandatory for a common pathway to be differentially regulated by same set of regulators under different conditions. For instance, oncogenic pathways like “signaling by notch” and “cancer module 252” are regulated differentially by a variable set of regulators under different conditions. The results not only support the pathway-centric approach in elucidating conserved elements for cancer diagnosis, but also reassures robustness of TF based algorithms in detection of key elements governing biological processes.

In order to get a list of statistically significant altered pathways, we also measured alteration score for each APA predicted altered pathway from 1000 shuffled case-control datasets (see Supplementary Method). We observed 170 significantly altered pathways (FDR adj. p ≤ 0.05) in PDC which included *PPARA* mediated pathways along with cancer related pathways like “p53 signaling pathway” and “signaling by notch”. We observed that around 70% of the altered pathways also undergo TF mediated differential regulation. Intriguingly, among the significant differentially regulated pathways, “PPARA activates gene expression” pathway exhibits maximum alterations in terms of connection type and strength. The results further reiterate the importance of *PPARA* in distinguishing prostate cancer samples from the normal ones. For complete list of pathways and its associated p-values, kindly refer to the Supplementary File S2.

Another unique APA feature is its ability to calculate the enrichment of context-specific disease genes within the predicted altered pathways. We analyzed disease gene enrichment score for predicted altered pathways in the PDC dataset. For reference, we included 106 known disease seed genes for identification of novel disease genes by Random Walk by Restart (RWR) algorithm^35^. The seed genes were obtained via data mining approach and found to act as driver genes in more than 12 cancer types (see Materials and Methods). The maximum disease enrichment score was obtained for cancer-related pathways, which is expected, as known cancer genes were selected as seeds. We observed elevated disease gene enrichment scores among the highly altered pathways for pathway related to focal adhesion kinase-mediated signaling events. We observed that amongst the top 10 disease genes-enriched pathways, 6 were cancer related, including prostate cancer pathway. Interestingly, most of the disease gene enriched pathways were comparatively less altered than the rest of predicted pathways. However, we failed to predict significant enrichment of disease genes associated within “PPAR signaling pathway”.

## Discussion

Different methodologies have exploited alteration in gene-gene relation or co-expression to predict functional gene set with alternately connected network profiles. However, not all altered pathways have gene components that are differentially regulated under influence of TF(s) and most of the existing methodologies ignore such functional gene sets. In APA, we classified the altered pathways as differentially regulated in which one or more DE genes share rewired interaction with its known TF. Herein, we assumed that only those TF-TG rewiring are regulatory and effective that lead to the differential expression of TG without altering baseline TF expression. Use of regulatory relationship to predict pathway alteration was first adopted by netGSA; however, it does not distinctly include TF and its upregulated TGs for prediction of differentially regulated pathways^14^. However, APA offers a unique data integration approach to search pre-defined regulatory interactions in a pathway sub-network with differential behavior across case-control matrices. Moreover, unlike other pathway analysis methods, APA uses only shortlisted rewired edges in a pathway sub-network for prediction of pathway alterations. These features elucidate a unique list of prioritized pathways not reported by many other pathway analysis tools. Although APA methodology is designed to integrate both rewiring effect and regulatory interaction to predict pathway alteration, gene connection rewiring is a mandatory requirement within pathway sub-network for APA, irrespective of the number of target genes differentially expressed in a given gene set.

In order to validate and evaluate APA performance, we performed APA analysis of p53 status dataset, which included expression profiles of 33 cell line samples with mutated TP53 gene. Since TP53 is a TF, any algorithm should readily detect aberration in p53 dependent pathways. Therefore, we hypothesized the alterations in direct molecular targets of the p53 gene as a result of its mutation. However, the pathway analysis methods evaluated by us failed to predict the “p53 signaling pathway” or pathways directly linked to p53 perturbation. However, APA readily predicts p53 dependent pathways with significant scores. In a nutshell, overall results provided necessary evidence that *TP53* mutation has profound consequences on gene expression regulatory profile. We observed common features demonstrated by most cancer genes, including p53 thereby suggesting connection rewiring to be an essential property associated with disease genes driving disease progression. The presence of several cancer-linked pathways with numerous oncogenic connections clearly suggests that TF-mediated rewiring is an important phenomenon that governs a disease progression and APA can effectively identify such interactions. Since APA algorithm distinctly emphasizes on the regulatory edges and other rewired edges, APA prioritized functional gene sets are different from the existing methodologies. The aforementioned unique APA analysis also complements traditional methods such as ORA for novel drug target discovery and may also be exploited for diagnostics and prognostic research.

Using APA, we also scanned the altered pathways in pancreatic cancer microarray expression dataset. We observed significant transcriptional changes in pathways like “E2F transcriptional network”, which plays a significant role in cell cycle progression, apoptosis, DNA damage and DNA repair mechanism^36^. *E2F1* is not only significantly rewired but it is also the most central pathway gene in the global rewired network. This suggests that rewiring analysis along with TF-associated differential regulations is important for determination of elements involved in pathway alteration. Apart from obvious and cancer-associated pathways, pathway alterations mediated by a nuclear family of transcription factors, i.e. *PPAR*, displayed a highly disturbed expression profile. The pathway is reported to act on several biological processes, including inflammation, lipid metabolism, adipogenesis, and maintenance of metabolic homeostasis^31^,^32^,^33^. The pathway also plays a crucial role in cancer-development and progression, though its role in pancreatic cancer progression is still not clear and mechanistic action yet to be elucidated. Since PPAR and many other pathways were not reported in previous studies, and in order to reaffirm the APA results, we performed APA analysis on two independent PDC datasets. We observed 141 common altered pathways, including *PPARA* mediated signaling pathway too. The results indicate that a common altered pathway is composed of dissimilar gene sets controlled by a diverse set of regulators. The findings are in agreement with previous studies indicating that pathway-based approach was better than gene-based approach for sample classification and elucidation of conserved features in heterogonous gene expression datasets, such as those of cancer. Apart from human cancer datasets, APA may also be used for analysis of altered pathways using gene expression datasets of different species, representing different contrasting conditions.

Summarily, APA detects altered gene-centric and edge-centric features rendering unique capabilities to elucidate novel altered gene sets. APA also ranks dysfunctional gene set on the basis of its enrichment with network-specific genes of interest, which can potentially aid researchers to shortlist key pathways important in alteration.

## Conclusion

To best of our knowledge, APA is first tool of its kind which computes gene network rewiring using gene expression datasets and performs comparative deep analysis for prioritization of differentially regulated and altered pathways. The unique APA features allow users to convert case-control gene expression datasets into testable hypotheses. The APA pipeline also renders pathway prioritization by context-specific altered pathways with higher precision, on both real as well as simulated datasets. Another novelty in APA is its ability to re-prioritize the altered pathways with data driven disease gene enrichment within rewired sub-networks. APA not only identifies the causal regulator and altered interactions in target genes, it also performs gene prioritization within each altered pathway with abnormal network properties.

## Materials and Methods

The methodology for dataset preparation for APA analysis is discussed in Supplementary Method.

### Construction of rewired gene co-expression networks

APA algorithm relies on a pair of undirected graphs generated from gene expression levels of case-control samples. An undirected graph is represented as ordered pair *G* = (*V, E*) in which a set of vertices *(V)* is connected by a set of edges *(E)*. Using the common space, APA constructs two such graphs, one each for case and control samples; using the Pearson Correlation Coefficient (PCC) to calculate the relationship between each pair of genes. Prior to generation of correlation graphs, the tool removes genes with no variability in expression level (σ_X_ = 0.00) to restrict the computation of false positive interactions. The differential expression of a gene is calculated with a linear modeling approach using limma R package^37^. For subsequent analysis, APA uses a single graph in which each edge represents the strength of gene pair association calculated by measuring the differences in edge strengths across two graphs i.e. edge rewiring.

Simulation based studies have previously shown that Fisher’s transformation of correlation strength (*r*) can significantly improve prediction accuracy of rewired gene edges^38^. Therefore, the tool first transforms each correlation coefficient using the following equation:


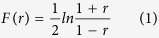


After the transformation, APA calculates Fisher’s test of difference between two corresponding edge strengths from both graphs. The test statistics considers change in correlation strength as well as effect of sample size (*n*) and it approximately follows standard normal distribution when the null hypothesis shows no difference between edge correlation strength amid case and control conditions. Fisher’s test of difference to compute rewiring strength of each gene pair is performed using the following equation:


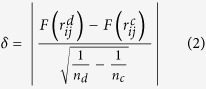


where, 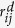 and 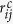 represent the correlation strength between a pair of genes (*i* and *j*) in control and disease sample, respectively. Next, APA measures the relationship of each *rewire* score to mean value, which represent rewiring strength, in a set of scores using the following equation:


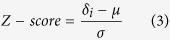


where *δ*_*i*_ represents rewiring score of *i*^th^ edge in the disease network; μ represent mean value of the scores set and σ represent its standard deviation. Finally, APA converts each edge Z-score to a corresponding p-value using two-tailed test of population proportion. Now edges with False Discovery Rate (FDR) adjusted p-value ≤ 0.05 were considered as significantly rewired. In the final rewired graph, APA assigns edge score by:


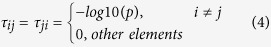


where the parameter *p* is p-value representing edge strength of any edge except self-loop.

### Construction of a rewired gene regulatory network

For constructing a rewired gene regulatory network, APA uses a square matrix Σ of size equal to number of vertices in the rewired graph in which edge elements are assigned by:


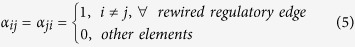


A rewired edge can be regarded as regulatory if at least one of the genes is a known TF which leads to up-regulation of its known TG (FDR adjusted p-value ≤ 0.05). To achieve this task APA uses a background gene regulatory network composed of predicted as well as experimentally validated interactions. We exploited three different data sources, namely TTRUST, RegNetwork and TcoF-DB, to generate pre-compiled list of regulatory edges which together host 22408 interactions among 1024 TFs and 5571 TGs^39^,^40^,^41^. For rest of the edges, including self-loops, APA assigns zero values to square matrix Σ.

### Altered pathway Identification

To identify aberration associated with each gene set or pathway, the tool uses a pre-compiled list of pathway terms and its associated gene components from several different sources. The user-defined pathway terms can also be analyzed using APA (please refer to the APA user manual). Pathway information related to KEGG^42^ and BIOCARTA (www.biocarta.com) were obtained from MsigDB along with 431 cancer modules from its C4 collection^43^, whereas, pathway information for Reactome (dbRC, http://www.reactome.org/, R package reactome.db version 1.54.1, http://bioconductor.org/packages/reactome.db/) and Panther (R package panther.db version 1.0.3, http://bioconductor.org/packages/PANTHER.db/)^44^ were obtained from their respective Bioconductor annotation packages^45^,^46^. We also collected pathway information from NCI-PID which hosts a list of cancer related curated pathway terms and gene sets^47^. We converted gene IDs in all the pathways to corresponding ENTREZ IDs and obsolete IDs were either converted to a newer version or removed from the gene set. We removed pathway terms in which gene sets do not have minimum number of genes (*n* < 6); and among redundant pathway terms only, the ones with bigger gene sets were retained. Finally, the remaining 2,057 pathway terms comprising of 13,638 genes were retained for case-specific altered pathway analysis. Users can also input an alternate list of pathways in a APA acceptable format (details are mentioned in the online manual). However, it is mandatory that ID format must be consistent across pathway gene sets and gene expression datasets.

Next, APA assesses whether a given pathway gene set is enriched with a significant number of rewired genes. To measure significance, APA maps gene set of each pathway term onto the rewired network and computes the number of rewired genes enriched within a pathway gene set. For each pathway gene set, statistical significance of gene overlaps is measured using Fisher’s exact test-generated p-value, corrected by Benjamini-Hochberg algorithm (FDR adjusted p-value ≤ 0.05). Among the enriched gene set of a pathway term, APA identifies number of TGs/TFs differentially regulated under influence of its known TF(s). This is achieved either via local or global regulatory rewired connection(s), depending on if TF itself is a component of the pathway. Differential regulation score (*Dr*) for each pathway gene set *pw* is then computed by measuring average fold change in expression of all the target genes that share rewired edge with known TF(s). The differential regulation score is then combined with average rewiring score to compute pathway alteration score, i.e. Dy. In algebraic terms, normalized alteration ‘*Dy*’ score for each altered pathway is computed as follows:


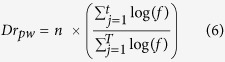



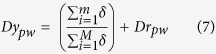



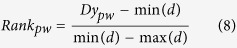


where *δ* represents rewiring score of *i*^th^ edge in rewired sub-network of a given pathway *pw,* having *m* number of rewired edges within *M* connections among *T* genes. Here, *n* represents number of regulatory rewired edges associated with *j*^th^ gene among *t* number of target genes having an expression fold change *f* across two conditions. Here *d* represents set of alteration scores predicted for all pathway gene sets with at least one rewired edge.

### Disease gene enrichment in altered pathway

APA offers two different algorithms for measuring connectivity association between known disease genes and other vertices in a rewired network. Both the algorithms work in similar fashion and require a subset of seed gene IDs that are part of a rewired network. Therefore, using literature-search we shortlisted 106 genes that were known to perform tumour related functions in 12 or more cancer types^48^,^49^. We reassured the functional oncogenic significance of 106 seed genes by mapping each gene on the TAG database which hosts known tumour-associated genes^50^.

To identify context-specific disease genes, the tool computes affinity score for each network gene by applying RWR algorithm (default method)^35^. Briefly RWR is an iterative walker that displays a transition from specific seed node (e.g. *TP53*) to a random neighbor with a probability *r*. Equation for RWR is defined as:


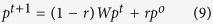


where, *p*^*t*^ denotes affinity vector having size equal to vertex count in a rewired graph and *i*^th^ element has probability to be at node *i* at a time step *t*; *W* is column normalized (intra-array normalization) adjacency matrix of rewired graph. The tool also assigns an initial equal probability (*p*^*o*^) to each seed node representing known disease genes such that sum of the probabilities equal to one. Similarly, affinity score for each graph vertex can be computed using k_node_ based method which is thoroughly discussed in ref. 51. The total disease gene enrichment score (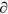) for each altered pathway is calculated by APA, using the following equation:


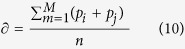


where *p*_*i*_ and *p*_j_ denote affinity score of *i*th and *j*th genes forming an intra-pathway rewired connection in a given altered pathway with *M* connections. The tool uses RWR as implemented in dnet R package^52^.

### Calculation of gene network properties

For identification of most central gene per pathway, APA measures four different network properties of each gene in rewired networks. The four network properties are closeness, transitivity, degree and page rank, calculated using igraph R package^53^. The rewired network includes only significantly altered edges and therefore centrality measures predicted for each gene represent differential gene attributes present in disease network rather than in control network. The network centrality score for each gene is calculated by the tool using following equation:


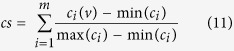


where *c*_*i*_*(v)* refers to centrality of a given gene and *m* = 4 refers to group of four centralities. Here *cs* measures overall centrality assigned to each gene in a global rewired network.

### Implementation

APA is designed as a cross-platform package, easily executable via command-line interface. APA source code is written in PERL and R programming languages and output is presented as a Cytoscape JavaScript generated DHTML report, for user-friendly sub-network visualization. Default input files for APA are designed to use background human data. However, background GRN and pathway database for non-human datasets can be transparently customized for APA use. APA user manual provides useful information about APA usage, including command line options for non-human dataset customizations.

## Additional Information

**How to cite this article:** Kaushik, A. *et al*. Altered Pathway Analyzer: A gene expression dataset analysis tool for identification and prioritization of differentially regulated and network rewired pathways. *Sci. Rep.* **7**, 40450; doi: 10.1038/srep40450 (2017).

**Publisher's note:** Springer Nature remains neutral with regard to jurisdictional claims in published maps and institutional affiliations.

## Supplementary Material

Supplementary Information

Supplementary Dataset S1

Supplementary Dataset S2
