# Supplementary material for: Altered Pathway Analyzer: A gene expression dataset analysis tool for identification and prioritization of differentially regulated and network rewired pathways
Source: Sci Rep. 2017 Jan 13;7:40450. doi: 10.1038/srep40450 (PMC5233954; doi:10.1038/srep40450)
Supplement: Supplementary Information [file srep40450-s1.pdf]

Altered Pathway Analyzer: A gene expression dataset analysis tool for identification and prioritization of differentially regulated and network rewired pathways.

Abhinav Kaushik[1], Shakir Ali[2], Dinesh Gupta[1*]

[1] Translational Bioinformatics Group, International Centre for Genetic Engineering and Biotechnology, New Delhi 110067, India

[2]Department of Biochemistry, Jamia Hamdard, Deemed University, New Delhi 110062, India.

* Corresponding author

email: dinesh@icgeb.res.in

Keywords: Altered pathway, gene network rewiring, gene expression dataset analysis, systems analysis.

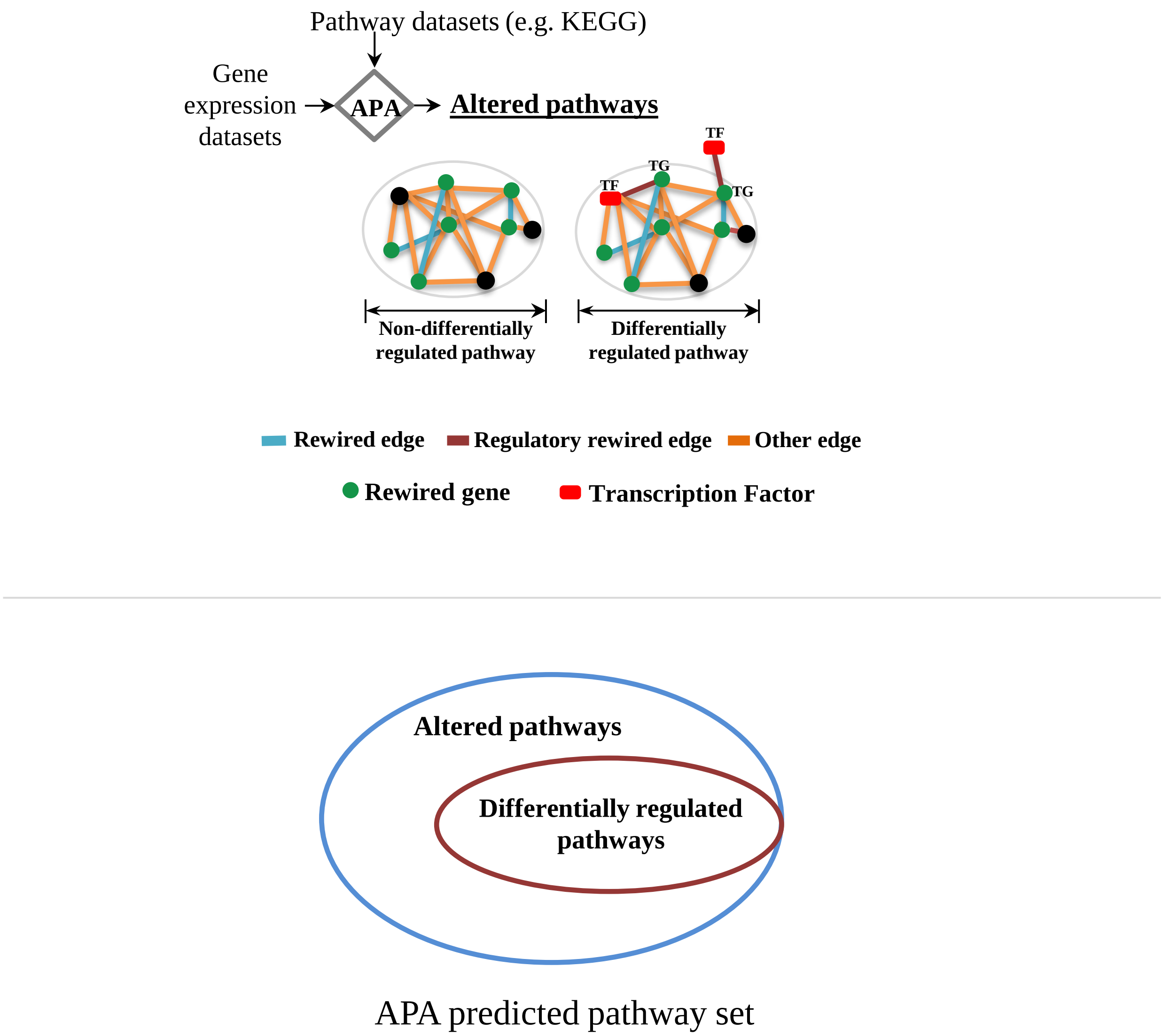

Supplementary Figure S1. Altered vs Differentially regulated pathway

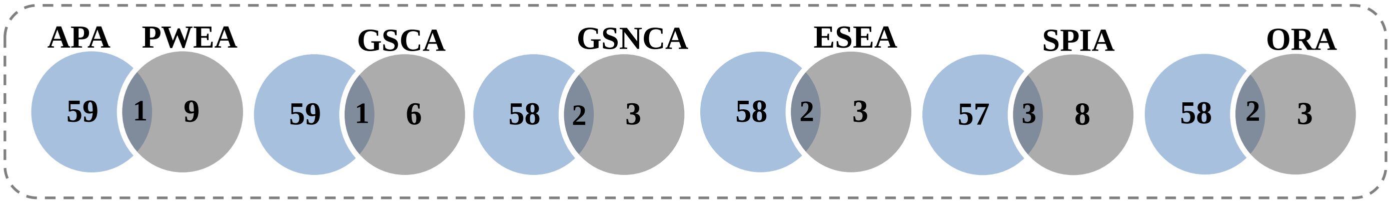

Supplementary Figure S2. Common altered p53 target pathways predicted with APA and other tools.

Supplementary Method

A. *Gene expression dataset processing*

The processed p53 status gene expression dataset from NCI-60 cell lines was obtained from GANPA dataset R package [1]. The dataset contains gene expression values for 10100 probes representing 50 different samples (17 TP53wildtype and 33 TP53 mutated). The genes in the dataset were represented by gene symbols, which were converted to entrez IDs, a mandatory requirement for running APA analysis.

As the first step in dataset processing, we obtained annotation file for each GEO dataset from its respective GPL series and mapped the probe IDs to corresponding entrez IDs. We observed that multiple probes were mapping on common entrez IDs. Therefore, we merged the gene expression levels of two or more rows with common gene entrez IDs using *collapserow()* function in WGCNA R package, using default parameters [2]. In the next step we attempted to perform inter-array normalization to obtain normalized expression level of all genes in each dataset with either 14 bit or 16 bit resolution [3]. However, we found that gene expression level in all the datasets used in this study were already within the limits and, therefore, not normalized.

B. *Measuring the statistically significance of altered pathways in prostate cancer*

The statistical significance of the predicted alteration in perturbed pathways was measured by randomly shuffling samples in the case-control datasets (e.g. p53 mutated and wild type samples). We repeated 1000 different iterations to obtain a random distribution in each dataset and for each shuffle total normalized score $d^*$of each altered pathway was obtained. The level of statistical significance for each altered pathway (P_{pw}) is evaluated as:

$$P_{pw} = \frac{\#\{d_{pw}^{*} \geq d_{pw}\}}{t}$$

Where $\#\{T\}$ is the cardinality of set T; and we controlled the Benjamini-Hoschberg FDR associated with each p-value P_{pw} for multiple hypothesis correction.

Supplementary Method. **A.** The detailed methodology used for preparing and processing the datasets before executing APA analysis. **B.** The methodology used to measure the statistical significance of altered pathways in prostate cancer.

References

1 Fang, Z., Tian, W. & Ji, H. A network-based gene-weighting approach for pathway analysis. *Cell research*. **22**, 565-580 (2012).

2 Langfelder, P. & Horvath, S. WGCNA: an R package for weighted correlation network analysis. *BMC Bioinformatics*. **9**, 559 (2008).

3 Heider, A. & Alt, R. virtualArray: a R/bioconductor package to merge raw data from different microarray platforms. *BMC Bioinformatics*. **14**, 75 (2013).

Supplementary Table S1. APA predicted statistically significant pathways in p53 dataset.	
**Pathway Name**	**P-value**
STEROID_HORMONE_BIOSYNTHESIS	0.006
MAPK_SIGNALING_PATHWAY	0
SPHINGOLIPID_METABOLISM	0
PATHWAYS_IN_CANCER	0.008
GLYCEROPHOSPHOLIPID_METABOLISM	0.037
RETINOL_METABOLISM	0
AMINO_SUGAR_AND_NUCLEOTIDE_SUGAR_METABOLISM	0
LONG_TERM_POTENTIATION	0.013
LYSINE_DEGRADATION	0
PYRUVATE_METABOLISM	0.016
